# Mapping of Female Breast Cancer Incidence and Mortality Rates to Socioeconomic Factors Cohort: Path Diagram Analysis

**DOI:** 10.3389/fpubh.2021.761023

**Published:** 2022-02-01

**Authors:** Qiongle Peng, Xiaoling Ren

**Affiliations:** ^1^Blood Transfusion Department, Affiliated Hospital of Jiangsu University, Zhenjiang, China; ^2^Central Laboratory, Wuxi Traditional Chinese Medicine Hospital, Wuxi, China

**Keywords:** mortality, breast cancer, incidence, socioeconomic factors, regression analysis, path diagram analysis

## Abstract

**Objectives:**

Breast cancer is the leading cause of death in women around the world. Its occurrence and development have been linked to genetic factors, living habits, health conditions, and socioeconomic factors. Comparisons of incidence and mortality rates of female breast cancer are useful approaches to define cancer-related socioeconomic disparities.

**Methods:**

This was a retrospective observational cohort study on breast cancer of women in several developed countries over 30 years. Effects of socioeconomic factors were analyzed using a path diagram method.

**Results:**

We found a positive, significant association of public wealth on incidence and mortality of breast cancer, and the path coefficients in the structural equations are −0.51 and −0.39, respectively. The unemployment rate (UR) is critical and the path coefficients are all 0.2. The path coefficients of individual economic wealth to the rates of breast cancer are 0.18 and 0.27, respectively.

**Conclusion:**

The influence of social pressure on the incidence and mortality of breast cancer was not typical monotonous. The survival rate of breast cancer determined by the ratio of mortality rate to incidence rate showed a similar pattern with socioeconomic factors.

## Introduction

Through history, health was always one of the most fundamental issues of human development. In most of the historical stages, the culture, economy, trade, and war experienced in each country were part of objective existence, and closely associated with individual health problems (1). Health problems faced by human beings were influenced by the following aspects (2). First, the time (3) and space (4) on which human beings depend for survival constitutes the objective basis for the development of human society (5). The second reason was the basic living necessities. Third, human health issues are strongly tied to special spatial conditions (6, 7), such as longitude, latitude, altitude, and temperature (8). These environmental factors will cause or induce people to form a life and behavior habit or culture (9) that matches the geographical conditions (10, 11). Fourth, human health problems tightly relate to social development (12–14). At different stages of development, human beings face different threats of diseases (3, 15). The types and severity of diseases were different in different regions and ethnic groups at the same historical stage (16). Hunger (17), disease (18), and death are the three basic threats for the objective existence of species (19), and the same is true of the advanced animals, i.e., human beings (20).

Today, cancers have brought a huge threat to human health. Human beings have made considerable progress in dealing with breast cancer, such as disease prevention (21), cancer screening (22), etiological analysis (23), targeted drugs (24, 25), and clinical surgery (26). Despite important advances in the understanding of oncogenesis and development in the past decades, breast cancer remains one of the most common cancers diagnosed among women and the leading cause of female cancer death (27). The risk of breast cancer was significantly increased in developing counties (28). The occurrence and development of breast cancer are complex and multi-stage processes, which prompt humankind to tackle at least two short- or long-term goals. On the one hand, it is urgent to develop new-targeted drugs or explore minimally invasive surgical techniques. On the other hand, it is important to consider the factors of breast cancer occurrence and development from the perspective of social/environment factors (29, 30) and their interactions (31) (e.g., support (32) and education, networks (33), and emotion (34)) on breast cancer occurrence and development. Numbers of individual and environmental factors may contribute to the risk of breast cancer and the prognosis in patients. Recently, the correlations between socioeconomic status and breast cancer incidence and mortality rates are increasingly recognized.

Studies have demonstrated that nature environmental, host genetic, and socioeconomic factors influence the breast cancer prevalence landscape with a far-reaching influence on racial disparity to subtypes of breast cancer (35). The socioeconomic effects on the incidence and death of the breast cancer need to pay enough attentions, the socio-economic disparities in breast cancer survival prevail in this relatively homogenous society (36). Note that the function of public wealth and individual wealth are different during the intervene process. Thus, the lower screening attendance for women with lower socioeconomic status, and higher socioeconomic status is linked to higher incidence but lower case fatality (37). Importantly, there is limited understanding of the contribution of social factors to control patterns (30). Further, the influence and interaction of many socioeconomic factors (e.g., disposable wealth and pressures of life) on breast cancer of women are complex, and it is difficult to expose the dominate factors by cutting off the cross effects of affecting factors. This study is critical for the influences of the socioeconomic factors on the development of breast cancer, with a purpose of providing socioeconomic information for the high-risk screening and diagnosis, prevention, and managing long-term surveillance care of female breast cancer. Besides, this study is useful for the instructive intervention of social welfare and public health policy, with consideration of the prevention and treatment on breast cancer of women in developed countries and regions.

## Methods

In this study, a retrospective observational cohort study on breast cancer of women in Denmark, Norway, Italy, New Zealand, Israel, France, Germany, and Japan between 1980 and 2012 was carried out. The regression analysis and multivariate analysis (path diagram analysis) for five factors, i.e., years, population, gross domestic product (GDP), gross domestic product per capita (GDPPC), and unemployment rate (UR), were adopted using Excel database function, and the effects of socioeconomic factors on breast cancer incidence and mortality rates were analyzed. The breast cancer incidence and mortality data from 1980 to 2012 were obtained from Global Cancer Observatory (GCO) (http://gco.iarc.fr/#cancer-overtime). The socioeconomic data (such as GDP, GDPPC, UR, and population) of several representative developed countries were obtained from National Accounts Main Aggregates Database. (https://unstats.un.org/unsd/snaama/Basic). An illustration of the incidence and mortality of breast cancer is provided in Supplementary Figure S1. For multi-factors problems, the multivariate multiple linear regression formula usually can be expressed as the sum function of intercept and the product of the variable and the partial regression coefficient. Using the regression equation of samples, the least square method was used to find coefficients to minimize the sum of squares (SS) of residual errors. The structure of path diagram analysis was represented by a series of regression parameters. Hypotheses involved the correlational and regression-like relations between the incidence/mortality rate and the socioeconomic factors. That is, some factors were observed variables and the others were latent variables. There might be a relationship between the observed variables and latent variables, and some variables maybe functions of other variables.

Path diagram analysis is a form of structural equation model (SEM) and is generally tested by regression analysis (38). The structural model is fitted by mathematical statistics methods and principles (39). After the series test and analysis, the most suitable model was available to represent multiple complex relationships between independent variables and variables. In this study, we focused on certain socioeconomic factors, such as time-dependent public wealth, living environment, including social population and unemployment ratio (40) (reads social pressure), and individual economic wealth. As well-known, these factors have complex interactions with each other. Therefore, path diagram analysis, based on multiple linear regression models, was used to explore the factors which influence female breast cancer using the multi-dimensional causality and related strength analysis (Supplementary Figure S2).

In this study, the sample includes information for numerical variables from several representative countries (e.g., Denmark, Norway, New Zealand, Canada, Israel, France, Germany, and Japan), such as information on economics and breast cancer during 1980–2012. The value of Ri (*i* = 1–5) is the stepwise route of the regression analysis. We assumed that the five factors (*x*_1_, *x*_2_, *x*_3_, *x*_4_, and *x*_5_) of years, population, GDP, GDPPC, and UR affect the dependent variable, i.e., the incidence and mortality rates (*y*_1_, *y*_2_) of breast cancer. Further, we supposed that time was the most basic variable, and GPD was a low-order variable. GDP as a function of social wealth was impacted by years, a measure of time, GDPPC, population, and UR, which were all high-order variables and affected by other variables (as shown in Supplementary Table S1). The survival rate of breast cancer was measured using the mortality-to-incidence ratio (MIR) (41) and to illustrate the effects of socioeconomic factors (42). All variables were normalized before regression analysis. In mathematical form, the regression equation of path diagram analysis was formed by linking these variables with some coefficients. During each process of regression, X_i_ is an independent variable, which consists of years, population, GDP, and GDPPC, and UR. *Y*_j_ (*j* = 1–5) is the dependent variable, in this case, the incidence rate and mortality rate of breast cancer. During the process of stepwise regression analysis, note that the identity of *X*_*i*_ (except for the lowest-order independent variable *X*_1_) would transform into a dependent variable *Y*_*j*_.

## Results

There is a significant relationship between breast cancer incidence and time and socioeconomic factors (i.e., population, GDP, GDPPC, and UR) (*R* > 0.8), except for factors identified by the stepwise regression model. The fifth regression analysis (for the model of population vs. time, *R* < 0.2), there are significant multi-factor correlations. This finding verifies that the model is reasonable. The main function of ANOVA table is used to judge the regression effect of regression model by joint hypotheses test (*F*-test). The ANOVA data of each step regression analysis is listed in Table 1. In the process of decreasing regression, the degree of freedom (*df* ) of potential variables is reduced by one for each regression. The significant level, or *F* statistics for each step of the regression analysis has different *p*, which is less than the stated significance level of 0.05. Therefore, the regression equation for each step has the statistical significance of regression process. Further, the obtained interception and partial regression coefficient are used to express each regression equation. The revalued coefficients for the selected models of the multivariable analyses are presented in Table 2.

**Table 1:** The ANOVA results of regression models for incidence rate.

**Step**	**Variation sources**	***df***	***SS***	***MS***	***F***	**Significance F**
I	Regression	5	4.0823	0.8164	47.8654	9.48E-36
	Residual	282	4.8101	0.0171		
	Total	287	8.8924			
II	Regression	4	17.1575	4.2894	102.9645	5.35E-54
	Residual	283	11.7895	0.0417		
	Total	287	28.9470			
III	Regression	3	3.5235	1.1745	44.5455	1.27E-23
	Residual	284	7.4880	0.0264		
	Total	287	11.0115			
IV	Regression	2	5.0085	2.5043	230.1414	3.23E-60
	Residual	285	3.1012	0.0109		
	Total	287	8.1097			
V	Regression	1	0.2594	0.2594	5.7892	0.0167
	Residual	286	12.8143	0.0448		
	Total	287	13.0737			

*Significance F (F significant statistic) has the p that is less than the significance level of 0.05, so the regression equation has a statistical significance*.

**Table 2:** A structural equations model based on the regression analysis of the incidence and mortality rates of female breast cancer.

**Step**		**Coefficients**	**Standard errors**	**T stat**	***P*-value**	**Lower 95%**	**Upper 95%**
I (Incidence)	Intercept	−20.208	2.0584	−9.8175	9.24E-20	−24.2597	−16.1563
	Year	20.7499	2.0960	9.8999	5.02E-20	16.6242	24.8756
	GDP	−0.5088	0.0550	−9.2508	5.74E-18	−0.6171	−0.4006
	GDPPC	0.1795	0.0616	2.9114	0.0039	0.0581	0.3008
	UR	0.1864	0.0371	5.0271	8.87E-07	0.1134	0.2594
	Population	0.1956	0.0282	6.9352	2.77E-11	0.1401	0.2511
I (Mortality)	Intercept	3.1114	2.7758	1.1209	0.2633	−2.3526	8.5755
	Year	−2.6725	2.8265	−0.9455	0.3452	−8.2364	2.8913
	GDP	−0.3906	0.0742	−5.2658	2.77E-7	−0.5366	−0.2446
	GDPPC	0.2654	0.0831	3.1927	0.0016	0.1018	0.4290
	UR	0.1967	0.0500	3.9332	0.0001	0.0983	0.2952
	Population	0.0170	0.0380	0.4475	0.6548	−0.0578	0.0919
II	Intercept	12.0378	4.2787	2.8134	0.0052	3.6157	20.4598
	Year	−12.1658	4.3577	−2.7918	0.0056	−20.7434	−3.5881
	GDP	1.3156	0.0856	15.3741	3.42E-39	1.1472	1.4841
	GDPPC	0.1652	0.1295	1.2755	0.2032	−0.0898	0.4202
	UR	0.3932	0.0746	5.2720	2.68E-07	0.2464	0.5400
III	Intercept	−23.3425	3.1094	−7.5072	7.84E-13	−29.4629	−17.2222
	Year	24.28957	3.1530	7.7036	2.22E-13	18.0833	30.4958
	GDP	−0.64	0.0565	−11.3269	8.63E-25	−0.7512	−0.5288
	GDPPC	−0.77568	0.0922	−8.4125	1.99E-15	−0.9572	−0.5942
IV	Intercept	−25.0936	1.3344	−18.8053	7.37E-52	−27.7201	−22.4671
	Year	25.5634	1.3453	19.0016	1.41E-52	22.9154	28.2114
	GDP	−0.3654	0.0291	−12.5382	5.13E-29	−0.42272	−0.3080
V	Intercept	−6.3643	2.6814	−2.3735	0.0183	−11.6422	−1.0864
	Year	6.5029	2.7027	2.4061	0.0168	1.1832	11.8227

*In this structural equations model, the dependent variables (i.e., incidence rate and mortality rate) have the highest ranking. We assume that the other variables are their independent variables no matter whether implied values or not. That is, the breast cancer incidence and mortality rates are no longer used as variables to explore structural equation models after first-order regression. The underlined values of p in the table indicate the mathematical relationships that are not of statistical significance*.

**Figure 1:**
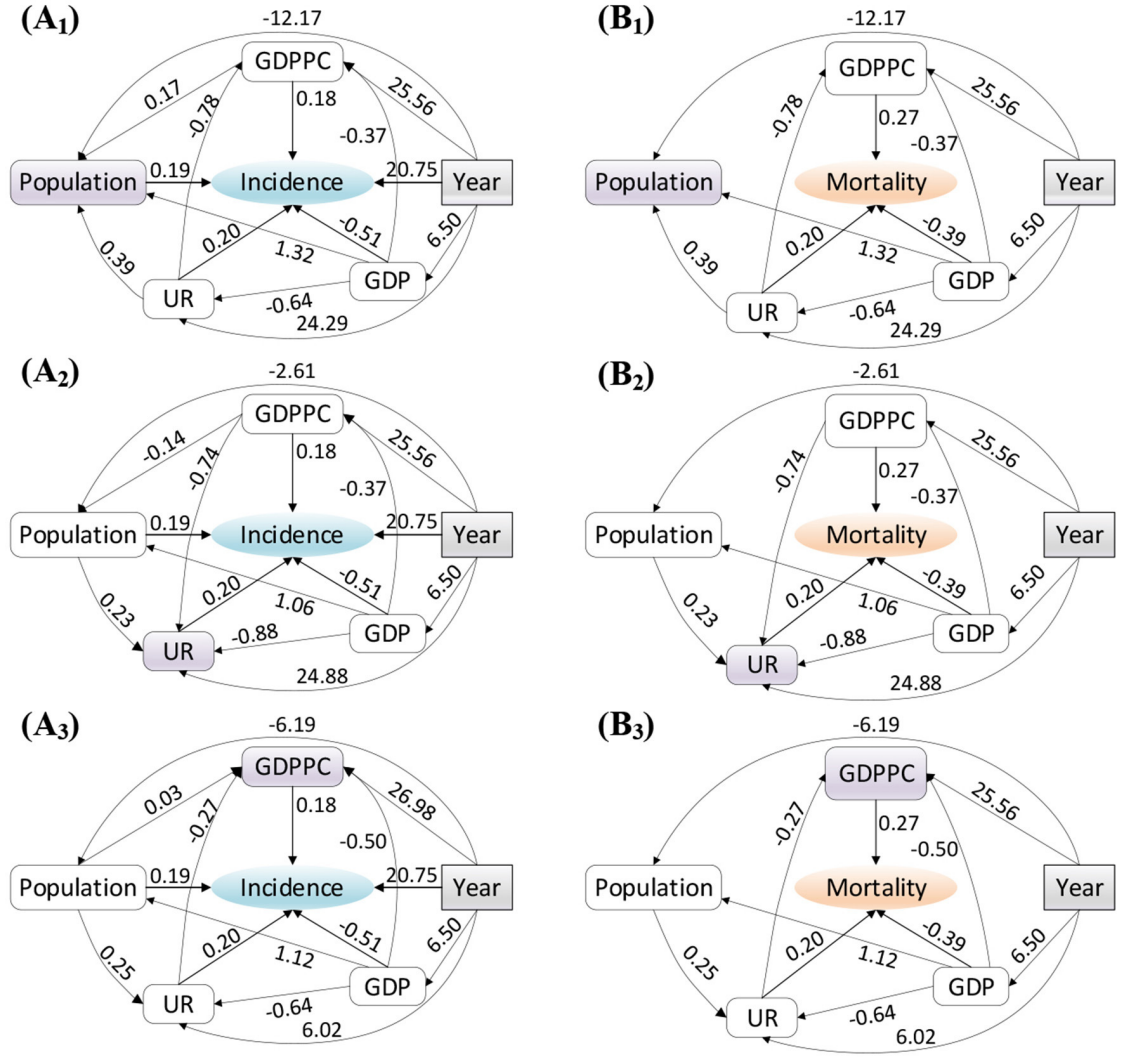
Structural equations model used to illustrate the relation between the **(A)** incidence and **(B)** mortality rates and socioeconomic factors. Among many variables, subscripts are used to represent high-order variables, of which subscript 1, 2, and 3 correspond to high-order variables population, UR and GDPPC respectively.

Note that the *p* of items of “intercept,” “year,” and “population” in the first regression are larger than the significance level of 0.05. The item of “GDPPC” in the second regression has no statistical significance (^**^p > 0.05). These shows that, the hypothesis that female breast cancer mortality is a function of time and population is not statistically significant in the structural equations model. In addition, the hypothesis that population is a function of GDPPC has no statistically significance. Therefore, some path coefficients in this structural equation models need eliminated for the reasonable hypothesis and correction SEM model. According to the path coefficients, we can understand and identify the cause–effect relationship between the latent variables. Further, the path diagram based on the structural equation models are obtained and as shown in Figure 1. In the path diagram model, the magnitude of the path coefficient indicates the relationship between the influence degree of variables and dependent variables, while the positive and negative values indicate the positive and negative effects of the influence trend.

Year was the most basic time variable which was always related to the incidence of breast cancer, regardless of which implied value, the path coefficient is the largest, as shown in Figure 1. The weight of mapping relationship was the largest, which ultimately led to the highest degree of impact on breast cancer incidence. In addition, social public wealth (GDP) has a greater impact on the incidence of breast cancer. Its negative value (−0.51) reflected that the incidence of breast cancer declines with the increase of GDP, which was benefited from the improvement of public health conditions, the development of medical technology, disease prevention and control propaganda, and other interventions. The influence of social pressure (UR), personal economic wealth (GDPPC), and population on the incidence of breast cancer is very close (the path coefficients are about 0.2). In different structured variance models, the positive and negative of path coefficients remain unchanged, but the values of path coefficients were different. These deep-seated socioeconomic relations were not discussed here.

**Figure 2:**
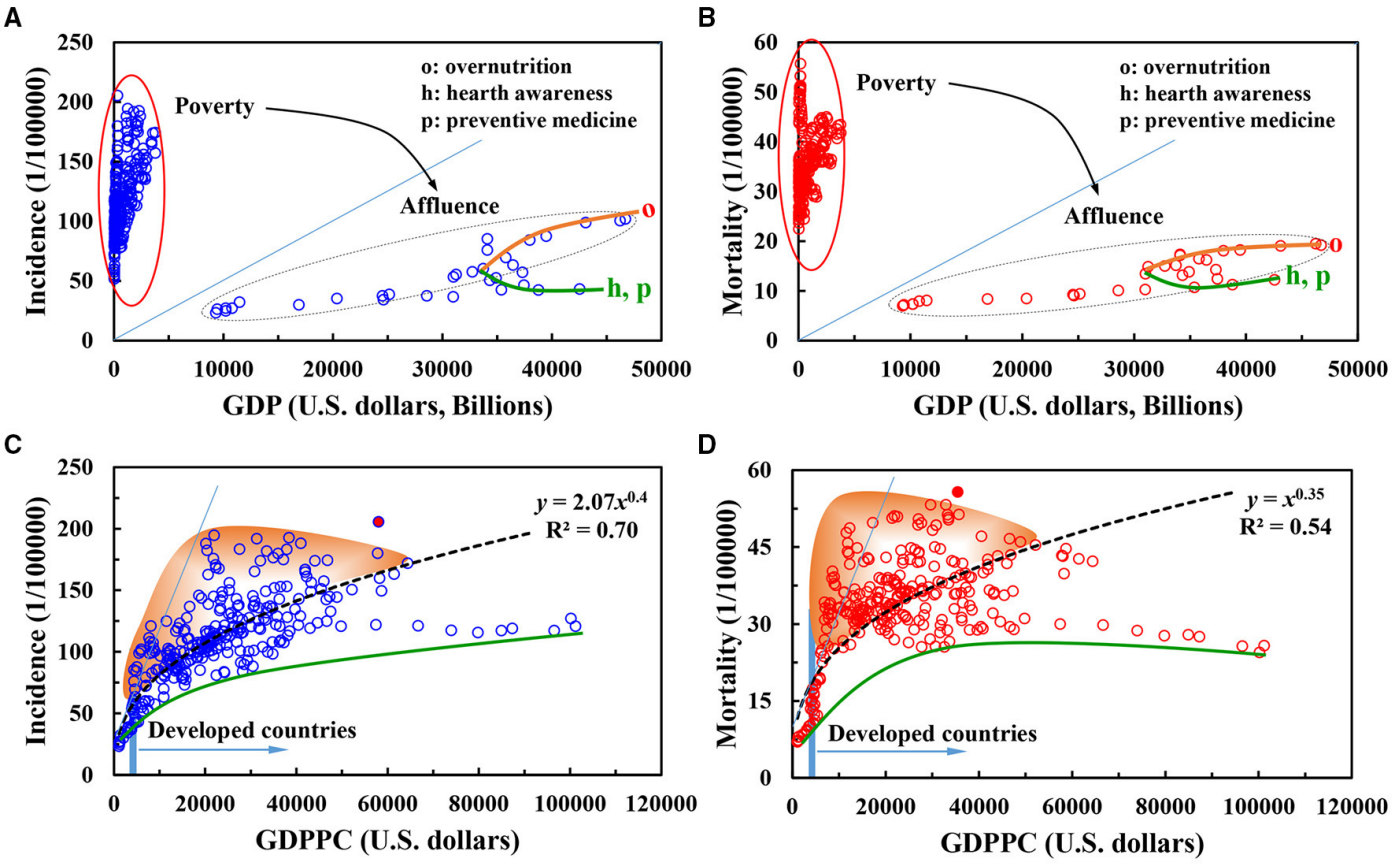
**(A–D)** Differentiated effects of social public wealth and per capita income level on incidence and mortality rates of female breast cancer.

However, the mortality and socioeconomic factors of patients with breast cancer are different from the incidence of breast cancer as shown in Figures 2A,B. The direct impact of year and population were not significant, and thus, we eliminated the two factors. The increase of public wealth helped to reduce the mortality rate of patients with breast cancer, but the increase of personal wealth (GDPPC) and social pressure (UR) induced the increase of mortality rate of patients with breast cancer. Therefore, reasonable control of personal economic wealth and release of social pressure are helpful to prolong the survival rate of patients with breast cancer. The path coefficients obtained by low-order regression are consistent with the incidence variables.

## Discussion

### Effects of Wealth Factors

The effect of country-independent GDP values on the incidence and mortality of breast cancer in women based on years were shown. As an important macroeconomic indicator, GDP best measured the economic strength and wealth of a country. The national GDP has an economic impact on the living standards and health of citizens. As seen, the influence of GDP on the incidence of diseases showed a significant separation phenomenon, and the effect of GDP on the mortality of diseases showed a significant separation phenomenon. At lower national GDP (i.e., the national economic status is in poverty), the incidence and mortality of female breast cancer were both higher. In addition, the incidence and mortality are highly concentrated in the range of 50–200 (per 100,000 persons) and 20–60 (per 100,000 persons). Under higher national GDP, where the national economic status was in rich and defining threshold was 10,000 billion, a good quasi-linear relationship between the incidence and mortality of female breast cancer and GDP was shown. When the GDP was between 10,000 and 30,000 billion, the incidence and mortality of female breast cancer increased slowly with the increase of GDP. This phenomenon might be related to the source of national wealth and industrial level. These factors might produce benefits to working women and work pressure, working environment, and labor intensity. When the GDP was >30,000 billion, the influence of GDP on the incidence and mortality of breast cancer shows a significant bifurcated separation phenomenon, such as “o” and “h/p” in Figures 2A,B.

There were possible reasons for this pattern. First, some samples reflected that the country were wealthy, where the living standard of people significantly improved. In these countries, the increasing incidence and mortality of breast cancer were related to over nutrition, obesity, and other problems associated with rapid economic development. In addition, in these countries, the working intensity of the people was surplus/deficiency, which led to the deviation of individual physique from the healthy range (43). Second, in other emerging economies, with the increase of GDP, the national investment in research and development of preventive medicine and medical technology was enhanced. The national awareness of disease prevention and health has significantly grown, resulting in a gradual decline in the incidence and mortality of breast cancer. Therefore, the low-income countries need to allocate sufficient resources to increase screening participation (44). Thereafter, it is available for the high quality of occurrence data and the adoption of accurate methods to estimate incidence and mortality.

As an important reference indicator for improving the per capita income level and living standard of residents, the GDPPC indirectly reflects the average purchasing power level of social individuals and the degree of independence of life. GDPPC was additionally used as an important economic index for individuals and families, and is related to the objective conditions of life and the judgment of the facts and values of the state (e.g., happiness index (45)). High GDPPC might enhance the individual happiness through the individual's independent, free, and pleasurable experience in life. The economic index reduces cancer incidence and mortality (46). In Figures 2C,D, the influence of per capita GDP on the incidence of female breast cancer and the trend of mortality have a power function change trend, but there is clear difference in the two key parameters of coefficient and power index. For example, the coefficient of the power function of incidence rate is two times the power function of mortality rate.

There were several reasons for this finding. First, women have a high degree of initiative and enthusiasm in the pursuit of personal value and economic wealth before the onset of breast cancer. At the same time, the increase in personal income, work stress, and work intensity was increased significantly, which results in an increase in the incidence of breast cancer. This result is different from the previous study. However, the effect on mortality is different. Overall mortality was greater among the patients with breast cancer of the lowest income group than in the highest one (47). When income is low, expensive medical expenses are major stressor for patients with breast cancer. The economic resources have a great impact on families. All aspects of stress will promote the negative beliefs in patients with breast cancer, further resulting in accelerated illness and death. This was represented in the upper part of the fitted curve. In wealthy families, the presence of patients with breast cancer will not put significant economic pressure on families or related members. After the pain and suffering caused by the disease, patients with breast cancer are willing to pay for better therapy and nursing. Furthermore, an open-minded attitude of life has reduced the mortality rate to some extent.

### Effects of Society Pressure

Sociological pressure is accompanied by every process of growth. Specifically, health deterioration from unemployment is likely to be large, and unemployment is a public health problem that needs more focus (48). Usually, an increase in the UR is a signal of economic weakness and a reflection of social pressure. For individual, the unemployment or insecure employment closely relates to the degree of happiness and social pressure, specifically psychological complaints, and life satisfaction. The impact of UR on individuals is reflected in psychological stress, which in turn affects breast cancer incidence and mortality of an individual. A 1% increase in unemployment is associated with a significant increase in colorectal cancer mortality in both men and women (49). As an important chronic disease, breast cancer was associated with the national UR on the affected individuals.

**Figure 3:**
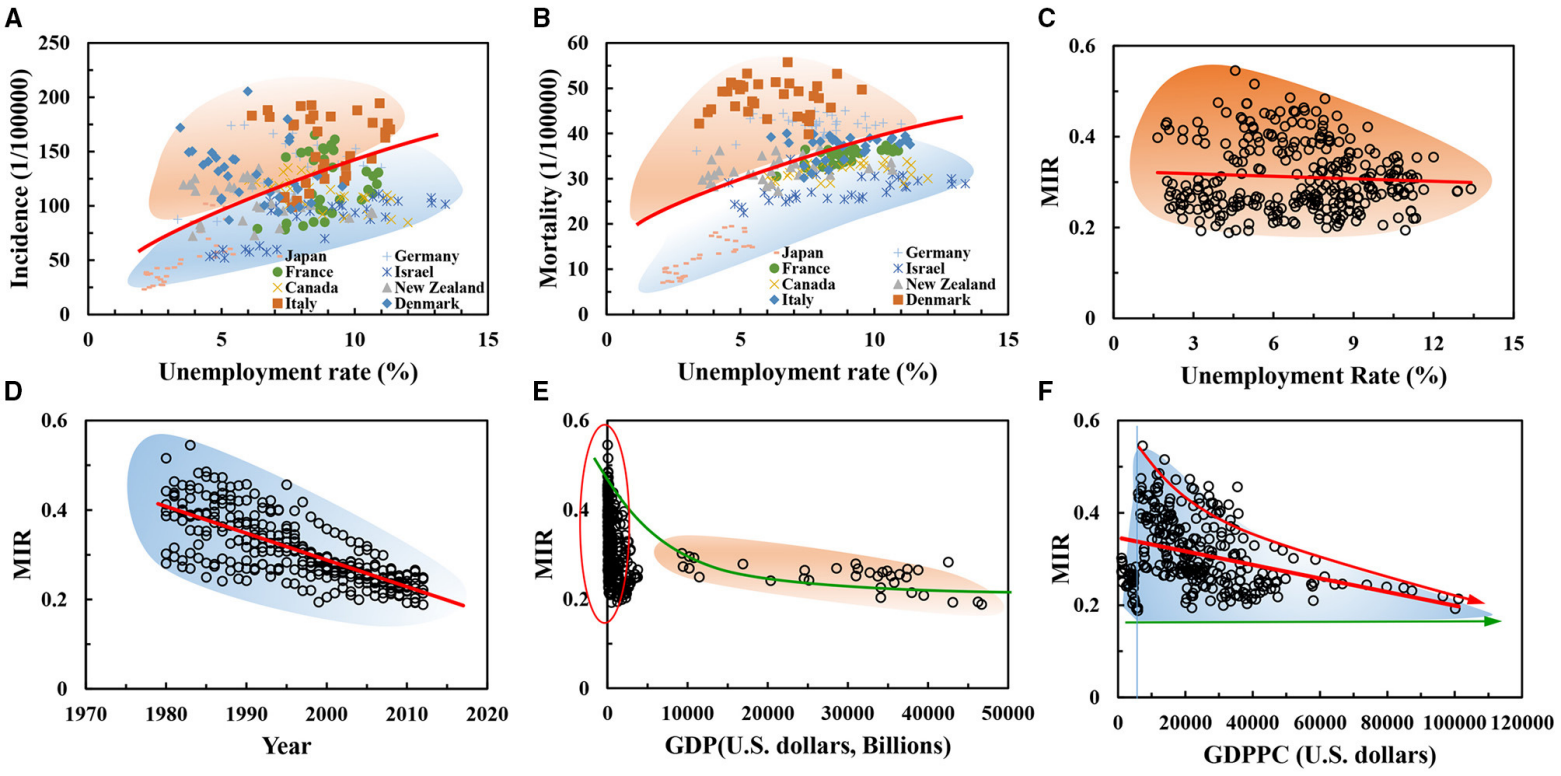
**(A–F)** Effects of socioeconomic factors on the mortality-to-incidence ratio (MIR) of female breast cancer.

Figure 3 shows the association of the country-independent UR on the incidence and mortality rates of female breast cancer. The UR has a reverse corresponding relationship with the economic growth rate, this trend is consistent to the structure equation analysis result. When the UR is too high, it impacts the income of unemployed group, and psychologically increases the insecurity of the unemployed. Some unemployed individuals may cause a series of problems in the case of poor psychological quality (50). This involuntary diffusion effect will increase the insecurity of workers in the industry, thereby increasing the overall insecurity of the society and having an important impact on the physical and mental health of individuals (51).

With the gradual increase of the UR, the incidence of breast cancer showed a growth trend of power function *y* = 42.27*x*^0.48^ (*R*^2^ = 0.30) Figure 3A; breast cancer mortality showed a power function *y* = 12.93*x*^0.48^ (*R*^2^ = 0.28) Figure 3B. Incidence and mortality have the same power exponent for the power function of independent variables, which can reflect the consistency of social pressure factors on individuals in the population (52). This consistency accounts for the dependence of human beings on social production relations and/or basic survival needs (53). These needs, which are of great importance to the quality of life and health factors of individuals (e.g., safety, food, and shelter), relate to the cognitive level of individuals themselves.

In addition, there is an approximate three-fold relationship between the coefficient of the incidence power function and the coefficient of the mortality power function, which relates to the individual's desire for life, health, and happiness (54). This result reads the 5-year survival rate (more than 60%), which is hoped to be a useful information for the patients of breast cancer. In this sense, external social pressure (such as, unemployment) might be fitted discretely in the curve of UR-incidence and mortality of breast cancer. However, the effects of unemployment are clear. Our results are broadly consistent with literature (55), unemployment significantly increases the risk of being dead at the end of follow-up by nearly 50%. The fact may ask for the deep think on the unemployment insurance system for the potential protective effects on the patients of breast cancer (50).

### On MIR

The 5-year survival rate of breast cancer usually is proxied by MIR of breast cancer for women health (42), Adams et al. investigated the accessibility and importance of mammography services (56). We further investigated the influences of four socioeconomic factors (such as, years, GDP, GDPPC, and UR) on MIR of female breast cancer, as shown in Figures 3C–F. In Figure 3, a sharp increase in the MIR of female patients with breast cancer was revealed with the increase of GDP and GDPPC from 1980 to 2012, see Figures 3E,F. MIR obeyed a power function from the trend of the power function of incidence and mortality aforementioned, but the correlation coefficient of regression analysis is small and the dispersion degree of data is high. However, the impact of the increased UR on MIR was almost constant, see Figure 3C. Therefore, the scatter plot shows that the differentiation was serious, which was mainly related to the physical quality, personal will, and survival belief of individual (57).

**Figure 4:**
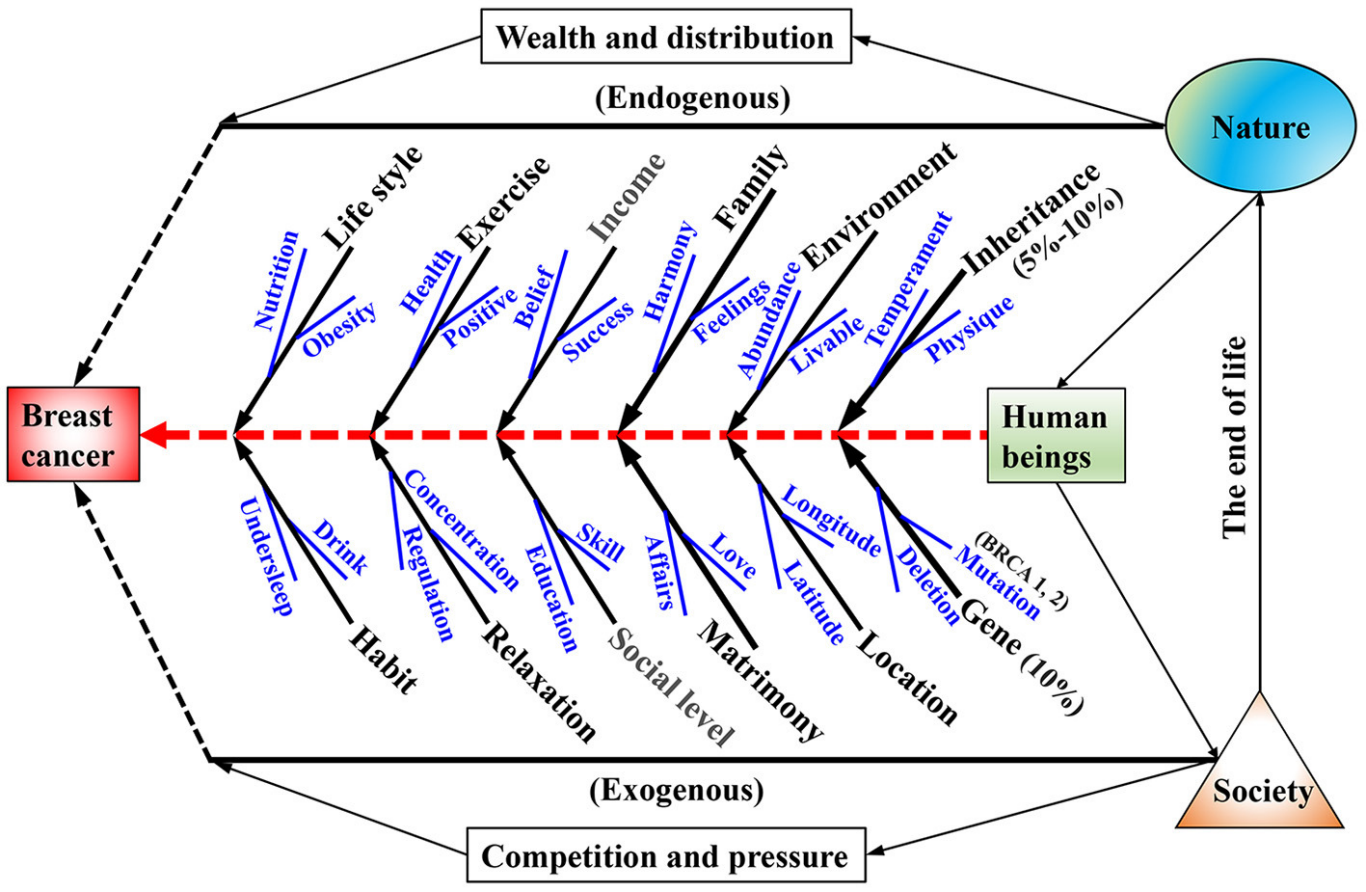
Fishbone diagram of the affecting factors both from nature and society on the female breast cancer.

As shown in Figure 4, the influence factors both from nature and society are various and are not available using a single way. The pathogenesis of breast cancer is not only related to the living environment, individual differences, the nature of work, and social roles, but also related to the socioeconomic factors of its comprehensive results. At present, there are still many unknowns in these studies, such as the structural coefficient between the factors and the influence weight. With the development of information network and the improvement of the accessibility of public statistics data sharing, there may be more connections between the factors that originally belong to the network, which need researchers to further carry out the research work of logical carding and model construction. Due to limited space, we cannot investigate the various factors from nature to society for breast cancer. To investigate the influence maps between the socioeconomic issues and breast cancer, we focused on two groups socioeconomic data in this study, one is endogenous wealth and distribution related to economic development (GDP and GDPPC), and the other is exogenous completion and pressure related to individual survive (UR and population). These factors were readily available and involved in cancer control, such as population-level incidence rates, death rates, and survival rates. Further, the socioeconomic factors based on development were investigated by using stepwise regression analysis and path diagram analysis.

## Conclusion

Social public wealth has a threshold limit on the regulation of breast cancer occurrence and development. The public wealth produces significant intervention ability until the value reaches at a certain level. The impact of social pressure (UR) on the incidence and mortality of female breast cancer was not typical monotonous but showed a power function trend in a specific range. Individual economic wealth has a strong intervention effect on the incidence and mortality of breast cancer. The survival index determined by the ratio of mortality to incidence showed a similar pattern with socioeconomic factors.

Bivariate analysis generally supported the results of univariate analysis. By using path coefficients and structured equations, the multivariable structured equation model analysis further accurately delineates the impact of socioeconomic factors on breast cancer incidence and mortality. The first-order structural equation model was subjected to socioeconomic factors, but the second-order structural equation model was related to the correlation between socioeconomic factors. Structural equation and path coefficient show that UR and personal wealth have important effects on the incidence rate and mortality of female breast cancer. On the one hand, doctors and hospitals can advise social forces to pay attention to and maintain a fair and warm social environment, on the other hand, they can appeal the government to consider the medical security mechanism for special groups and diseases (e.g., women breast cancer) in the allocation of public resources. In addition, the establishment and expression of mathematical models related to socioeconomic factors were of great value to the accurate analysis and quantitative prediction of the occurrence and development of breast cancer, and further provide an effective theoretical basis for the prevention and treatment of female breast cancer.

## Data Availability Statement

The original contributions presented in the study are included in the article/Supplementary Material, further inquiries can be directed to the corresponding author.

## Author Contributions

QP: conceptualization, investigation, methodology, data curation, funding acquisition, and writing—original draft preparation. XR: software, validation, formal analysis, resources, visualization, and writing—review and editing. All authors have read and agreed to the published version of the manuscript.

## Funding

This work was supported by Science and Technology program of Affiliated Hospital of Jiangsu University (No. jdfyRC2017003) to QP.

## Conflict of Interest

The authors declare that the research was conducted in the absence of any commercial or financial relationships that could be construed as a potential conflict of interest.

## Publisher's Note

All claims expressed in this article are solely those of the authors and do not necessarily represent those of their affiliated organizations, or those of the publisher, the editors and the reviewers. Any product that may be evaluated in this article, or claim that may be made by its manufacturer, is not guaranteed or endorsed by the publisher.
