# Supplementary material for: Mapping of Female Breast Cancer Incidence and Mortality Rates to Socioeconomic Factors Cohort: Path Diagram Analysis
Source: Front Public Health. 2022 Feb 1;9:761023. doi: 10.3389/fpubh.2021.761023 (PMC8843849; doi:10.3389/fpubh.2021.761023)
Supplement: Supplementary file 1 [file Data_Sheet_1.docx]

## Supplementary file

The multivariate multiple linear regression formula was expressed as follows:

$$\mu_{Y}=\beta_{0}+\beta_{1}X_{1}+\ldots\beta_{5}X_{5} (1)$$

where *β*_0_ is intercept, *β*_1_, *β*_2_…*β*_5_ is partial regression coefficient. By fitting the regression equation of samples $\hat{Y}=a+b_{1}X_{1}+\ldots b_{5}X_{5}$, and the least square method was used to find coefficients to minimize the sum of squares (SS) of residual errors.

$$SS=\sum_{i=1}^{5} \left( Y_{i}-a-b_{1}X_{i1}-b_{2}X_{i2}\ldots-b_{5}X_{i5} \right)^{2} (2)$$

The structure of path diagram analysis was represented by a series of regression parameters, as seen in Table S1. Hypotheses involved the correlational and regression-like relations between the incidence/mortality rate and the socioeconomic factors. That is, some factors were observed variables and the others were latent variables. There might be a relationship between the observed variables and latent variables, and some variables maybe functions of other variables. In this study, we used incidence and mortality rates as dependent variable. Generally, temporal and spatial variables were regarded as the most basic independent variable, and the other factors were function of time. A graphical explanation of the process of path diagram analysis and the raw five-factor model was shown in Figure S2.

Figure S1. Illustration of incidence and mortality of breast cancer of women in given developed countries after GCO data.

Figure S2. Graphical procedures and structural model for path diagram analysis.

Path analysis is one form of structural equations model (SEM), which initial was applied in econometrics. The attractive feature is that uses the structural equations to present the implied values and compare them with the observed values [1]. That is, it explicitly assumes that every variable we observe is an imperfect measure of some potential causal variables and that the causality of interests is always between these potential variables. During the modeling of structural equations, there are three matrices (**A**, **S**, and **F**) which are defined by McArdle and McDonald [2]. The so-called structural equations consist of these matrices, as expressed by$C=F\left( I-A \right)^{-1}S{(I-A)}^{-1'}F^{'}$. Where matrix **A** contains paths for asymmetric relations, **S** contains correlations and residual variances for symmetric relations, and matrix **F** filter the observed variables from the total data. Therefore, path diagram analysis is interesting and allow for novel applications [1, 3, 4]. In this study, it is possible to form three path analysis models, according to the previous assumptions about high-order variables (i.e. implied values) and low-order variables (i.e. observed values). The correlation coefficients and determination coefficients of the regression analysis of these three models are shown in Table S2.

Table S1. Regression sketch of numerical variable for the path diagram analysis.

| Route | Regression parameters | | | | | | Function |
| --- | --- | --- | --- | --- | --- | --- | --- |
| R_1_ | *X*_1_ | *X*_2_ | *X*_3_ | *X*_4_ | *X*_5_ | *Y*_1_ | *Y*_1_=*f*_1_(*X*_1_,*X*_2_,…*X*_5_) |
| R_2_ | *X*_1_ | *X*_2_ | *X*_3_ | *X*_4_ | *Y*_2_ |  | *Y*_2_=*f*_2_(X_1_,X_2_,…X_4_) |
| R_3_ | *X*_1_ | *X*_2_ | *X*_3_ | *Y*_3_ |  |  | *Y*_3_=*f*_3_(X_1_,X_2_,X_3_) |
| R_4_ | *X*_1_ | *X*_2_ | *Y*_4_ |  |  |  | *Y*_4_=*f*_4_(*X*_1_,*X*_2_) |
| R_5_ | *X*_1_ | *Y*_5_ |  |  |  |  | *Y*_5_=*f*_5_(*X*_1_) |

Note: The sample includes information for numerical variables from several representative countries (e.g. Denmark, Norway, New Zealand, Canada, Israel, France, Germany, Japan), including information on economics and breast cancer during 1980-2012. *R_i_* (*i*=1-5) is the stepwise route of the regression analysis. During each process of regression, *X*_i_ is an independent variable, which consists of years, population, gross domestic product (GDP), and gross domestic product per capita (GDPPC), and unemployment rate (UR). *Y*_j_ (*j*=1-5) is the dependent variable, in this case, the incidence rate and mortality rate of breast cancer. During the process of stepwise regression analysis, note that the identity of *X*_i_ (except for the lowest-order independent variable *X*_1_) would transform into a dependent variable *Y*_j_.

Table S2. Model summary for the multivariable regression analysis.

| **Model** | **Regression statistics** | **Step I** | **Step II** | **Step III** | **Step IV** | **Step V** |
| --- | --- | --- | --- | --- | --- | --- |
| Model 1 | Multiple R | 0.8389 | 0.7698 | 0.5657 | 0.7858 | 0.1408 |
|  | R Square | 0.7038 | 0.5927 | 0.3199 | 0.6176 | 0.0198 |
|  | Adjusted R Square | 0.6986 | 0.5869 | 0.3128 | 0.6149 | 0.0164 |
|  | Std. Error | 0.0968 | 0.2041 | 0.1624 | 0.1043 | 0.2116 |
| Model 2 | Multiple R | 0.6171 | 0.7434 | 0.7859 | 0.7858 | 0.1408 |
|  | R Square | 0.3808 | 0.5527 | 0.6176 | 0.6176 | 0.0198 |
|  | Adjusted R Square | 0.3720 | 0.5480 | 0.6149 | 0.6149 | 0.0164 |
|  | Std. Error | 0.1552 | 0.2135 | 0.1043 | 0.1043 | 0.2116 |
| Model 3 | Multiple R | 0.8340 | 0.4751 | 0.7420 | 0.7858 | 0.1408 |
|  | R Square | 0.6956 | 0.2257 | 0.5506 | 0.6176 | 0.0198 |
|  | Adjusted R Square | 0.6913 | 0.2175 | 0.5474 | 0.6149 | 0.0164 |
|  | Std. Error | 0.0934 | 0.1733 | 0.2137 | 0.1043 | 0.2116 |

Note: Complex correlation coefficient *R* is used to measure the degree of correlation between independent variables *x* and *y*. Complex determination coefficient *R*^2^ ($R^{2}=1-SS(\mathrm{residual})/SS(\mathrm{all})$) is used to explain the degree of variation of dependent variable *y* with the independent variable *x*, i.e. determine the fitting effect of dependent variable *y*. The adjusted complex coefficient *R*^2^ ($R_{adj}^{2}=1-\frac{MS(\mathrm{residual})}{MS(\mathrm{all})}$) can reflect the percentage of independent variable influencing dependent variable. Std. Error is used to measure the degree of fitting. The sample size is 288 in this study.

**Supplementary references**

[1] S.M. Boker, J.J. McArdle, M. Neale, An algorithm for the hierarchical organization of path diagrams and calculation of components of expected covariance, Structural Equation Modeling: A Multidisciplinary Journal, 9 (2002) 174-194.

[2] J.J. McArdle, R.P. McDonald, Some algebraic properties of the Reticular Action Model for moment structures, 37 (1984) 234-251.

[3] K.J. Grimm, N. Ram, Latent growth and dynamic structural equation models, Annual Review of Clinical Psychology, 14 (2018) 55-89.

[4] A.M. Abubakar, M. Ilkan, Impact of online WOM on destination trust and intention to travel: A medical tourism perspective, Journal of Destination Marketing & Management, 5 (2016) 192-201.
